# Turnover of Glycerolipid Metabolite Pool and Seed Viability

**DOI:** 10.3390/ijms19051417

**Published:** 2018-05-09

**Authors:** Xiao-Long Hu, Xiao-Mei Yu, Hong-Ying Chen, Wei-Qi Li

**Affiliations:** 1 Key Laboratory for Plant Diversity and Biogeography of East Asia, Kunming Institute of Botany, Chinese Academy of Sciences, Kunming 650201, China; huxiaolong@mail.kib.ac.cn (X.-L.H.); yuxiaomei@mail.kiz.ac.cn (X.-M.Y.); h.chen@mail.kib.ac.cn (H.-Y.C.); 2 University of Chinese Academy of Sciences, Beijing 100039, China; 3 The Germplasm Bank of Wild Species, Kunming Institute of Botany, Chinese Academy of Sciences, Kunming 650201, China

**Keywords:** hydration–dehydration cycle, membrane lipids, seed viability, phosphatidic acid (PA), diacylglycerol (DAG)

## Abstract

Hydration–dehydration cycles can frequently cause stress to seeds, but can also be used to improve germination. However, the molecular basis of the stress caused is poorly understood. Herein, we examine the effects of hydration–dehydration cycles on seed viability and profile the membrane glycerolipid molecular species. We find that seed viability was not affected during the first two cycles, but significantly decreased as further cycles were applied, until all viability was lost. The abundances of seven glycerolipid classes increased and decreased through hydration and dehydration, respectively, but the phosphatidic acid and diacylglycerol abundances changed in the opposite sense, while total glycerolipid contents remained constant. This suggests that during hydration–dehydration cycles, turnover of glycerolipid metabolite pools take place, while no significant lipid synthesis or degradation is involved. As further hydration–dehydration cycles occurred, lipid unsaturation increased, plastidic lipids decreased, and phosphatidylserine acyl chains lengthened. The latter two could be lethal for seeds. Our findings reveal a novel model of membrane lipid changes, and provide new insights into the responses of seeds to hydration–dehydration cycles.

## 1. Introduction

Seeds are not able to germinate without sufficient water under natural conditions [1,2]. However, sporadic rainfall and evaporation can cause seeds to experience hydration–dehydration cycles before germination [3,4]. Also, dew deposition at night and evaporation in the day cause diurnal hydration–dehydration cycles [5]. The seeds of desert plants are particularly likely to experience hydration–dehydration cycles, being frequently subjected to wetting–drying periods on the desert surface [6,7]. Single hydration–dehydration cycles are used artificially to improve seed germination in agricultural and forestry systems. Priming (semi-hydrating dry seeds and then dehydrating them) enables rapid and uniform germination [8,9,10], and can prevent imbibitional chilling injuries [11]. Survival or resistance to the effects of multiple hydration–dehydration cycles is an important trait in seeds both in the natural environment and in agricultural systems.

Seed hydration–dehydration cycles (differing in duration and number) have been investigated intensively at the physiological level. Approaches include soaking–drying, moisture equilibration–drying, and moist sand conditioning–drying [12]. Hydration–dehydration cycles can maintain or enhance seed vigor and viability [6,13,14,15], improve seed storability, and improve resistance to some stressors (e.g., heat shock and salt stress) [12,16,17,18,19]. In addition, wetting and drying cycles can also break seed dormancy [20]. However, hydration–dehydration cycles also cause membrane leakage and oxidative stress [21]. Long-term hydration or large numbers of cycles do not benefit and can even damage seed quality [15,22,23]. Thus, distinct hydration–dehydration treatments can positively or negatively affect germination.

**Figure 1:**
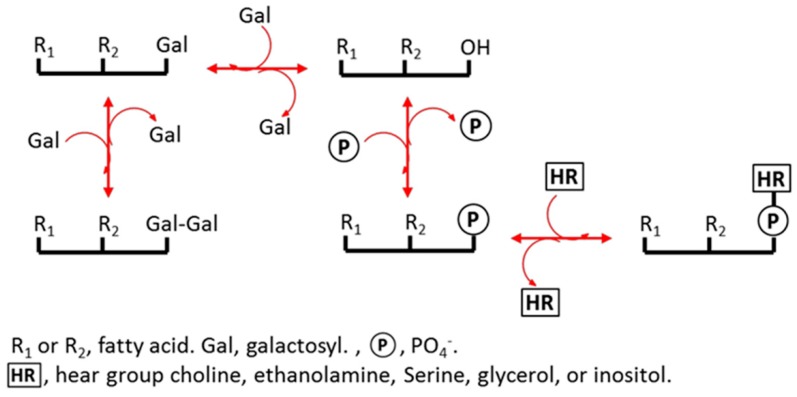
Schematic of glycerolipid turnover.

Plant membrane lipids are sensitive to changes in water content [24,25,26,27,28,29]. The components of plant membranes are mainly glycerolipids (see Figure 1 and Table S1), phosphatidic acid (PA), phosphatidylcholine (PC), phosphatidylethanolamine (PE), phosphatidylglycerol (PG), phosphatidylinositol (PI), phosphatidylserine (PS), digalactosyldiacylglycerol (DGDG), and monogalactosyldiacylglycerol (MGDG). In the glycerolipid synthesis pathway, membrane lipids are derived from the intermediate PA and diacylglycerol (DAG) at the plastid and endoplasmic reticulum (ER) (Figure 1). Environmental stress can cause the turnover of membrane lipids between each other to adjust local membrane features. For example, PA can be produced from PC and PE under freezing, mechanical wounding or drought conditions [28,30,31] because PA is cone-shape and fits the hexagonal II phase of membranes that have shrunk due to water deficit [11,32,33]. Therefore, PA content could be very high in mature dry seeds [11,34] and is known to decrease after seed imbibition [11]. A cellular water deficit commonly causes the degree of unsaturation of membrane lipids to increase [29,35]. A high degree of unsaturation increases membrane fluidity and can, therefore, maintain the physiological efficiency of a membrane [35,36]. Stress can also cause the acyl chain length (ACL) of PS to increase. The existence of long ACLs is speculated to be a signal of plant death [37]. However, it is not yet clear how membrane lipids in seeds respond to hydration–dehydration cycles.

We subjected seeds to hydration–dehydration cycles and profiled the molecular species in membrane lipids of *Arabidopsis thaliana* seeds using electrospray ionization tandem mass spectrometry, with the aim of determining how the seed membranes respond to continual changes in water content [31,38,39]. The amounts of all the membrane lipids, except PA, increased dramatically when the seeds were hydrated, and decreased when the seeds were dehydrated. The amount of PA present changed in the opposite direction, and in the same way as DAG. The total amount of glycerolipids present remained constant, but the proportion of PA plus DAG over other glycerolipids changed during the hydration–dehydration cycles. The degree of unsaturation of the membrane lipids changed and the ACL of PS increased as the number of hydration–dehydration cycles increased. Changes in lyso-phospholipids were also examined. We found novel metabolic changes in glycerolipids in the seeds in response to hydration–dehydration cycles.

## 2. Results

### 2.1. The Viability of Seeds Gradually Decreased as the Number of Hydration–Dehydration Cycles Increased

**Figure 2:**
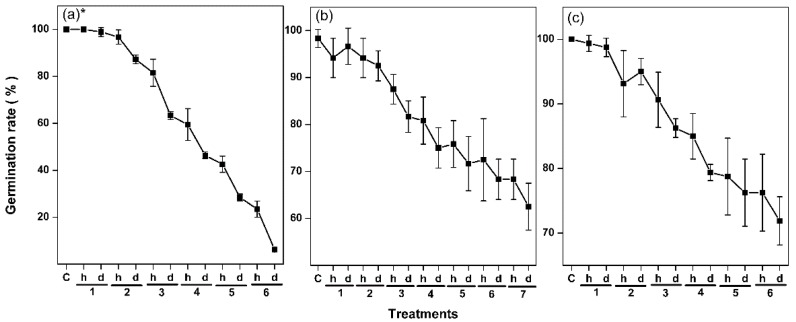
Effects of hydration–dehydration cycles on the germination levels of (**a**) *A. thaliana* seeds, (**b**) *L. perenne* seeds, and (**c**) *N. tabacum* seeds. The *A. thaliana* and *L. perenne* seeds were hydrated for 2 d and dehydrated for 5 d in each cycle, and the *N. tabacum* seeds were hydrated for 3 d and dehydrated for 5 d in each cycle. Germination data are mean ± SD (*n* = 4 replicates, each replicate being 30 or 40 seeds). h: hydration, d: dehydration. * The *A. thaliana* germination levels were published previously [34].

We first tested the effects of hydration–dehydration cycles on the viability of *Arabidopsis thaliana*, *Lolium perenne*, and *Nicotiana tabacum* seeds. We hydrated and then dehydrated seeds for six or seven cycles (Figure 2), and determined the germination level after each cycle. The germinations of three species seeds were different, which could be due to their distinct tolerance to dehydration, but they decreased markedly, from almost 100% to 6% for *A. thaliana*, from almost 100% to 62% for *L. perenne*, and from almost 100% to 73% for *N. tabacum*. The most significant decreases occurred after the second cycle for all three species. Hydration and dehydration both decreased the germination level, but dehydration had a stronger effect. These results show that hydration–dehydration cycles decrease seed viability, and that marked damage may occur after the second cycle. *A. thaliana* seeds are more sensitive than the others to the hydration–dehydration cycle, and provide the duration from full to almost no viability, so we used *A. thaliana* seeds in subsequent experiments.

### 2.2. The Total Membrane Lipid Contents of A. thaliana Seeds Changed Significantly through the Hydration–Dehydration Cycles

**Figure 3:**
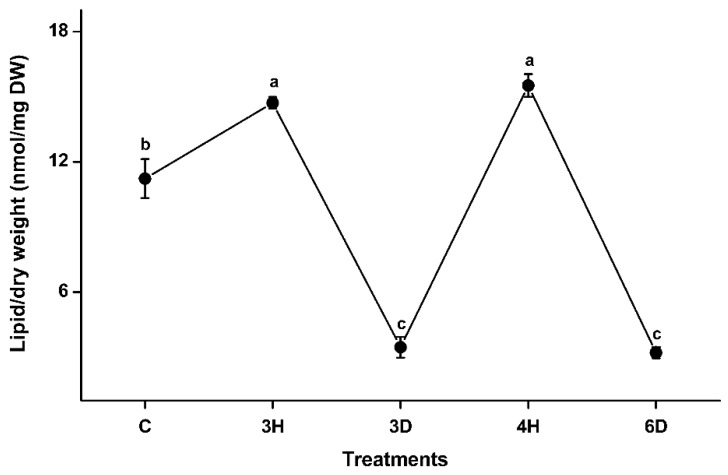
Total membrane lipid contents of *A. thaliana* seeds during the hydration–dehydration cycles. C: control (dry seeds), 3H: hydration part of the third hydration–dehydration cycle, 3D: dehydration part of the third hydration–dehydration cycle, 4H: hydration part of the fourth hydration–dehydration cycle, 6D: dehydration part of the sixth hydration–dehydration cycle. DW, dry weight. Each value is mean ± SD (*n* = 4 or 5). Values with different letters indicate that values are significantly different (*p* < 0.05).

Samples of *A. thaliana* seeds collected at five representative time points were subjected to lipidomics analysis. About 140 of the molecular species found in membranes were detected; these included six phospholipid classes (PG, PC, PE, PI, PS, and PA) and two galactolipid classes (MGDG and DGDG). The total membrane lipid contents changed significantly as the number of hydration–dehydration cycles increased (Figure 3 and Table S2). The total lipid content of the dry seeds (C) was 11.23 nmol/mg dry weight (DW). The lipid content increased in the 3H seeds by 31% (from 11.23 to 14.72 nmol/mg DW), then decreased >4-fold, to 3.46 nmol/mg DW, in the 3D seeds. The lipid content then increased by a factor of 4.5 (from 3.46 to 15.52 nmol/mg DW) in the 4H seeds, which was similar to the lipid content of the 3H seeds. The lipid content of the 6D seeds was almost five times lower than the lipid content of the 4H seeds, which was similar to the lipid content of the 3D seeds. These results indicated that hydration increased and dehydration decreased the membrane lipid contents in seeds. In other words, the membrane lipid content was positively related to the dynamic changed water content of the seeds.

### 2.3. Changes in Lipid Classes during the Hydration–Dehydration Cycles

**Figure 4:**
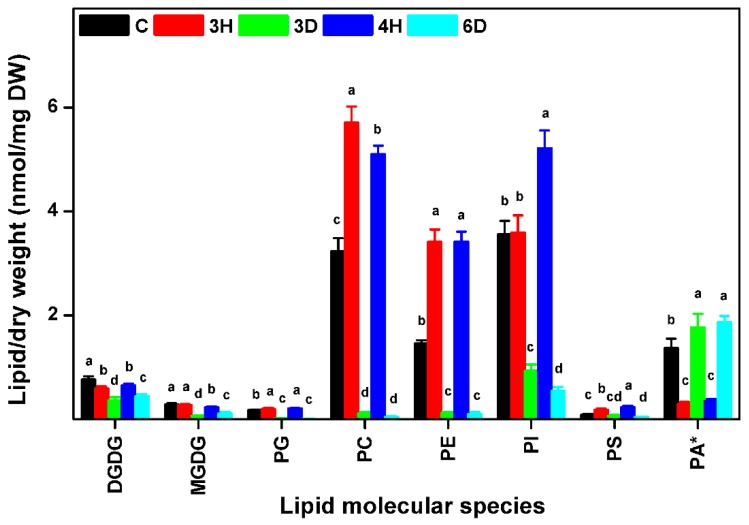
Amounts of membrane lipids in each head-group class in *A. thaliana* seeds during hydration–dehydration cycles. The treatment was the same as that described in Figure 3. DGDG, digalactosyldiacylglycerol. MGDG, monogalactosyldiacylglycerol. PG, phosphatidylglycerol. PC, phosphatidylcholine. PE, phosphatidylethanolamine. PI, phosphatidylinositol. PS, phosphatidylserine. PA, phosphatidic acid. DW, dry weight. Each value is mean ± SD (*n* = 4 or 5). Bars for the same lipid class with different letters indicate that the values were significantly different (*p* < 0.05). * The PA content was published previously [34].

**Figure 5:**
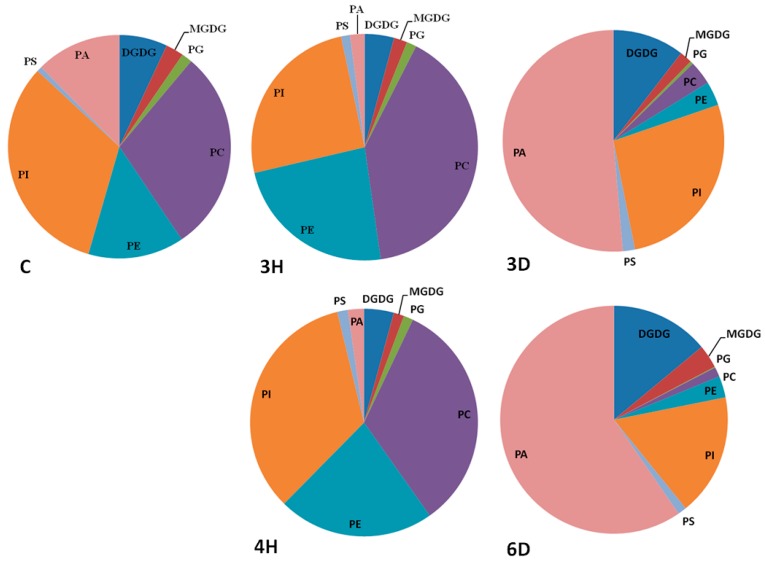
Contributions of the membrane lipids in each head-group class to the total membrane lipid contents of the *A. thaliana* seeds during the hydration–dehydration cycles. The treatment was the same as that described in Figure 3.

Different head groups give membrane lipids different physical and chemical properties that may affect their reactions to cellular water deficits. We investigated changes in each lipid class during the hydration–dehydration cycles, and found that the contents of all of the lipid classes, except PA, increased during hydration and decreased during dehydration, meaning that the contents of these seven lipid classes were positively related to the full hydration of the seeds (Figure 4 and Table S2). The PC, PE, and PI contents of the C seeds were higher than the contents of the 3D and 6D seeds, but lower than the contents of the 3H and 4H seeds. By contrast, PA followed the opposite trend to the other seven lipid classes (Figure 4). For example, the PA content of the C seeds was 1.37 nmol/mg DW, but the PA content of the 3H seeds was lower by a factor of 4.72 (from 1.37 to 0.29 nmol/mg DW). The PA content of the 3D seeds was 6.10 times higher (from 0.29 to 1.77 nmol/mg DW) to 3H seeds. These results indicated that specific membrane lipid classes in the seeds responded differently to the continuous hydration–dehydration cycles. The contents of seven lipid classes (PC, PE, PI, PG, PS, MGDG, and DGDG) positively related to full hydration, but the PA content was negatively related to full hydration. In addition, PA contributed >50% of the total lipid contents of 3D and 6D seeds (Figure 5 and Table S3). These results suggested that PA played a distinctive role in cell membranes during hydration–dehydration cycles, and even a critical role during the cumulative imposition of dehydration stress.

### 2.4. Downward Trends in MGDG and DGDG Contents during the Hydration–Dehydration Cycles

**Figure 6:**
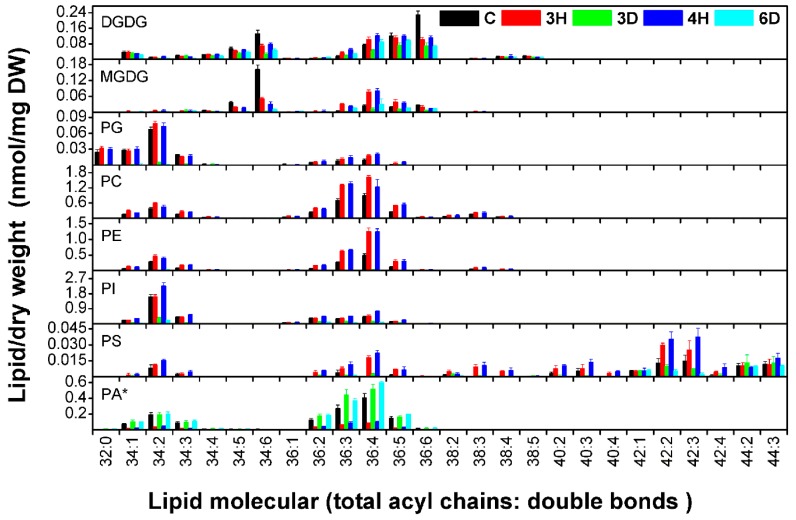
Molecular species contents of *A. thaliana* seeds during the hydration–dehydration cycles. The treatment was the same as that described in Figure 3. DW, dry weight. Each value is mean ± SD (*n* = 4 or 5). * The PA contents were published previously [34].

Each head-group class of membrane lipids contains many molecular species distinguished from each other by a standard (total acyl chains/double bonds). The contents and the compositions of almost 140 molecular species were shown in Figure 6 and Figure S1. These reflected the basic trends in the head-group classes, but differences between plastidic and extraplastidic lipids were found. The 34:6 and 36:6 molecular species were the main plastidic DGDG and MGDG species. The 34:6 and 36:6 molecule contents were significantly lower in the 3H and 4H seeds than in the C seeds, indicating that the plastidic lipid contents tended to decrease in seeds during hydration–dehydration cycles. Most of the extraplastidic molecular species of PC, PE, and PI followed the opposite trend, the lipid contents being significantly higher in the 3H and 4H seeds than in the C seeds. Decreasing the MGDG and DGDG contents could block plastid biogenesis [11], so the results supported seeds losing their viability after a number of hydration–dehydration cycles (Figure 2).

### 2.5. DAG and PA Mediated Glycerolipid Changes during the Hydration–Dehydration Cycles

**Figure 7:**
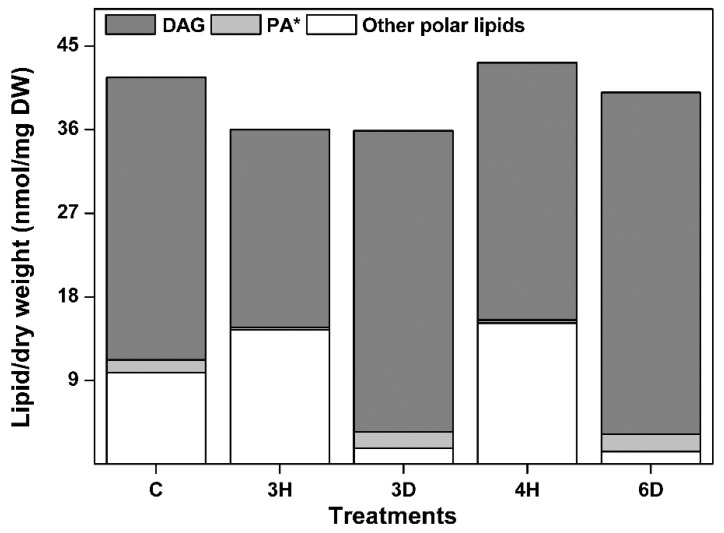
Total glycerolipid content of the *A. thaliana* seeds during the hydration–dehydration cycles. The treatment was the same as that described in Figure 3. DW, dry weight. * The PA contents were published previously [34].

Why did PA content change in the opposite way to the other lipid contents (Figure 4)? This could be due to its characteristics: (1) PA is a cone-shaped lipid, (2) PA is an intermediate in glycerolipid metabolic pathway. DAG is also cone-shaped and an intermediate in the glycerolipid metabolic pathway, so we propose that DAG may play the same roles as PA during hydration–dehydration cycles in seeds. To test this, we profiled the changes in DAG content during the hydration–dehydration cycles, and found that the DAG content was very high compared with the other lipid class contents, and that the DAG content followed the same trend as the PA content (Figure 7). These results indicated that the DAG content was positively affected by the drying phase of the hydration–dehydration cycles. This suggested that cone-shaped lipids and intermediates in glycerolipid metabolism may play important roles in the responses of seed membranes to dehydration.

**Figure 8:**
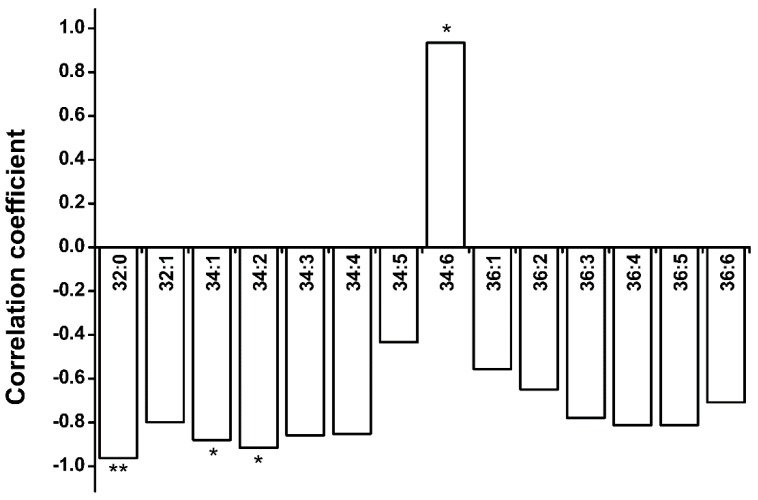
Correlation coefficients for group A and B of 14 molecular species. In group A, PA and DAG harbor all 14 molecular species. In group B, PG, PC, PE, and PI harbor 32:0 molecular species; PG, PE, and PI harbor 32:1 molecular species; DGDG, MGDG, PG, PC, PE, PI, and PS harbor 34:1, 34:2, 34:3, 34:4, 36:1, 36:2, 36:3, 36:4, 36:5, and 36:6 molecular species; DGDG and MGDG harbor 34:5 and 34:6 molecular species. The bars with asterisks are significantly different (* *p* < 0.05, ** *p* < 0.01).

Interestingly, the total contents of the glycerolipids that were measured, including DAG and the eight membrane lipid classes mentioned above, remained almost constant through the hydration–dehydration cycles (Figure 7), while the DAG and PA contents fluctuated dramatically. The variance of total glycerolipid content (10.4 ± 8.4%) was significantly lower than that of DAG plus PA content (36.6 ± 16.8%). This raised the question of whether the increases in DAG and PA (group A) content were caused by decreases in the contents of the other seven classes (group B), and vice versa. The turnover between two head-group classes could be investigated by examining the increase–decrease relationship between different molecular species with the same fatty acids [31]. We therefore performed correlation analyses of 14 molecular species in groups A and B (Figure 8) and found that 13 molecular species were negatively correlated, but the 34:6 molecular species were not. This suggests that turnover occurred between the 13 molecular species in groups A and B. Only MGDG, DGDG, PA, and DAG harbor 34:6 molecular species (Figure S2), and 34:6 MGDG and DGDG can be converted specifically into 34:6 PA under freezing conditions [39], so we performed correlation analyses on the 34:6 MGDG and PA, and on the 34:6 DGDG and PA contents (excluding the C seeds), and found negative correlations (*r* = −0.668 for 34:6 MGDG and PA and *r* = −0.772 for 34:6 DGDG and PA). This suggested that 34:6 MGDG and DGDG could be converted specifically into 34:6 PA during hydration–dehydration cycles. The results together suggested that the glycerolipid group A and B can convert into each other, but that the total glycerolipid content remained constant in the seeds during the hydration–dehydration cycles. In other words, cycling of glycerolipid metabolites take place during the hydration–dehydration cycles.

### 2.6. Membrane Lipids Became More Unsaturated during Dehydration

The double bond index (DBI) value represents the degree of lipid unsaturation [40,41]. We calculated DBI for the membrane lipids during the hydration–dehydration cycles, and found that for the total polar lipids, it changed significantly because of the cycles (Table 1). The DBIs for the C, 3H, and 4H seeds were 3.05, 3.05, and 3.02, respectively. The DBIs for the 3D and 6D seeds were 3.13 and 3.31, respectively, significantly higher than the DBIs for the C, 3H, and 4H seeds. This indicated that the unsaturation of the membrane lipids changed in response to seed drying.

### 2.7. The PS ACL Increased during the Hydration–Dehydration Cycles

**Figure 9:**
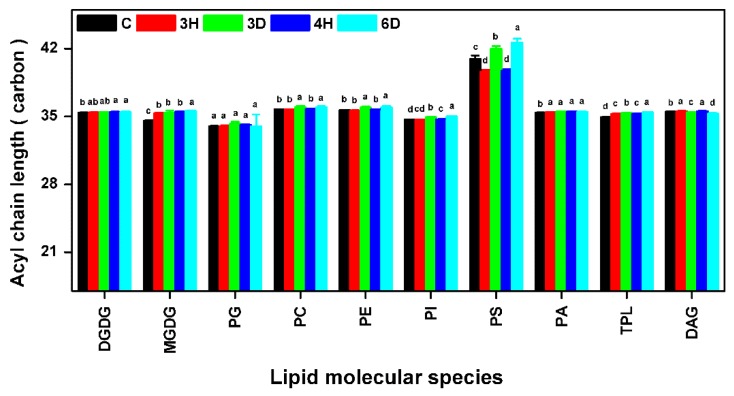
Acyl chain lengths (ACLs) of glycerolipids in the *A. thaliana* seeds during the hydration–dehydration cycles. The treatment was the same as that described in Figure 3. TPL, Total polar lipids. Each value is mean ± SD (*n* = 4 or 5). Bars for the same lipid class with different letters indicate that the values were significantly different (*p* < 0.05).

The lipid ACL can also affect membrane fluidity. We therefore calculated the membrane lipid ACLs, and found only slight changes except for the PS ACL (Figure 9 and Table S4). The mean membrane lipid ACL was 36 carbons, and the ACL changed by <0.13 carbon (<0.4%) during the continuous hydration–dehydration cycles. This suggested that the ACL contributed very little to membrane fluidity in the seeds during the hydration–dehydration cycles. By contrast, the PS ACL was significantly higher than the other lipid ACLs. The PS ACL increased significantly when the seeds were dehydrated and decreased when the seeds were hydrated. The PS ACL changed by 1.31 carbons (3.2%) between the C and 3H seeds, by 2.34 carbons (5.8%) between the 3H and 3D seeds, and by 2.89 carbons (7.6%) between the 4H and 6D seeds. The ACL of 6D seeds increased to 42.61 carbons. Similar results were previously found for leaves subjected to dehydration and heat shock [37].

### 2.8. The Profiling of Lyso-Phospholipids during Continuous Hydration–Dehydration Cycles

**Figure 10:**
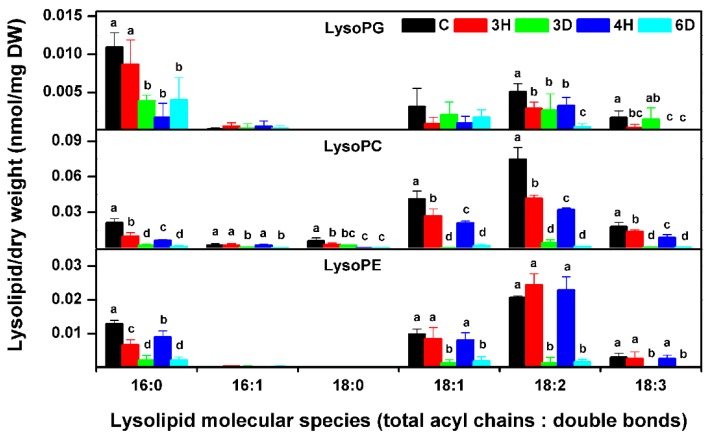
Lyso-phospholipid molecular species contents in the *A. thaliana* seeds during the hydration–dehydration cycles. The treatment was the same as that described in Figure 3. DW, dry weight. Each value is mean ± SD (*n* = 4 or 5). Bars for the same molecular species with different letters indicate that the values were significantly different (*p* < 0.05).

Lyso-phospholipids, including lysoPC, lysoPG, and lysoPE, are formed through the hydrolysis of fatty acid chain in phospholipids. Lyso-phospholipids are very sensitive to environmental stresses, such as freezing, cold, and heat shock [31,39]. We also profiled the lyso-phospholipid molecular species (Figure 10 and Table S5). The lysoPG content decreased over the full sequence of hydration–dehydration cycles. However, the lysoPC and lysoPE contents changed significantly during a single hydration–dehydration cycle, being highest for the C seeds, decreasing during dehydration, and increasing during hydration. These results indicated that there was different metabolism of specific lyso-phospholipids in seeds on moisture cycling.

## 3. Discussion

Seeds are commonly affected by hydration–dehydration cycles under natural conditions, and hydration–dehydration cycles have successfully been used to improve seed germination in agricultural and forestry systems. We confirmed the negative effects of multiple hydration–dehydration cycles on *A. thaliana*, *L. perenne*, and *N. tabacum* seed viability, and found that the germination level markedly decreased after the second hydration–dehydration cycle. We profiled the membrane glycerolipid molecular species in *A. thaliana* seeds during the hydration–dehydration cycles. The contents of seven classes of membrane lipids (PC, PE, PI, PS, PG, MDGD, and DGDG) in the seeds markedly increased and decreased as the moisture varied during hydration and dehydration, respectively, but the PA content changed in the opposite way. The content of the glycerolipid intermediate, DAG, changed markedly in the same way as the PA content. We found that the total glycerolipid content remained constant, even though the contents of PA plus DAG and the sum of other seven classes of glycerolipid changed markedly. These findings mean that there are metabolic cycles of glycerolipids during the hydration–dehydration cycles. Moreover, the plastidic lipid MGDG and DGDG contents decreased, but fluctuated, and the membrane lipid DBI and the PS ACL both increased. These results provided new insights into the lipid molecular responses of seeds to hydration–dehydration cycles.

The effects on seed viability had two distinct phases depending on the number of hydration–dehydration cycles. The first one or two cycles did not affect seed viability (Figure 2) and could even increase it if the cycles were carefully controlled, as in the agricultural practice called priming [9,10,42]. Seed viability gradually decreased, and was totally lost as more cycles occurred. In general, massive degradation of membrane lipids occurred in severe damaged leaves or dying plants [29]. It is puzzling that significant lipid degradation was not detected in *A. thaliana* seeds. even after the last hydration–dehydration cycle (Figure 4 and Figure 5), when viability was almost totally lost. However, indications of seed death were found in decreasing MGDG and DGDG contents (Figure 4 and Figure 6). Successful germination requires the plastidic lipid MGDG and DGDG contents to increase to allow plastids to form in the shoots [11]. Blocking MGDG and DGDG synthesis through hydration–dehydration cycles therefore prevents full germination. Another indication of seed death was the increase in the PS ACL (Figure 9), as PS acyl chains of living organs are limited to 42 carbons [37]. Thus, lengthening the PS acyl chains through hydration–dehydration cycles could cause seed death.

It is common for abiotic stress to induce changes in membrane lipids, particularly for the PA content to increase [29,43,44,45]. On the one hand, PA increase could be a signal for environmental stimuli [46,47,48], e.g., transiently because of mechanical wounding [45]. On the other hand, PA plays a role as a special membrane structure component with its cone-shape, such as its continuous and massive existence in membranes affected by drought or desiccation [27,29,34]. In this study, the role of PA appears to have been mainly structural, because it was produced in large quantities when the seeds suffered from water deficit and when the membranes shrank intensively. PA can be considered, at least partly, to be a glycerolipid metabolism intermediate, because the substantial turnover among these glycerolipids could occur through PA (Figure 1).

DAG is not a membrane lipid. It localizes in endoplasmic reticulum and plastids to serve as an intermediate in glycerolipid metabolism. The role of DAG in response to stress has been poorly studied. Our results showed, for the first time, that DAG was involved in the response of seeds to water stress. The DAG content of the seeds changed absolutely and relatively in a fluctuating manner during the hydration–dehydration cycles (Figure 7). It followed that the maintenance of total glycerolipids content would only result from an opposite fluctuation in the content of the other seven glycerolipid classes. In other words, (possibly not limited to the one we tested) seeds use internal cycles in the glycerolipid metabolite “pool” that do not involve synthesis and degradation to respond to hydration–dehydration cycles. This is probably an energy- and material-efficient strategy for dealing with environmental stress. But why do seeds decrease their membrane lipids under dehydration? We speculated that water deficit-induced cell shrinking causes its membrane area to decrease. The spare membrane lipids are converted into intermediate DAG. Under hydration, cell shape recovers through the reincorporation of functional membranes, including DAG. These bidirectional turnovers between internal glycerolipids can be performed quickly and efficiently as the seed moisture varies.

**Figure 11:**
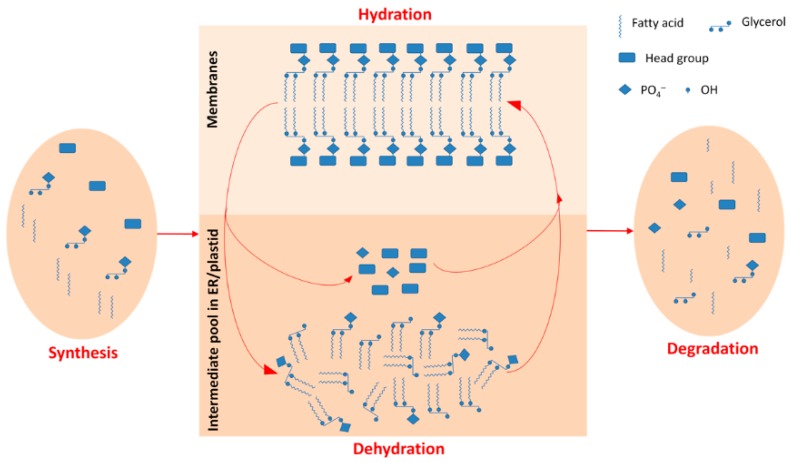
Working model for a metabolic cycle in glycerolipids during hydration–dehydration cycles. The metabolic cycles in glycerolipids are just between intermediate pool in endoplasmic reticulum /plastid and final products of glycerolipids in membranes and not involved in synthesis or degradation. The seeds use the metabolic cycles to respond to hydration–dehydration cycles.

In summary, our results indicated the mechanism through which hydration–dehydration cycles cause seeds to die. We proposed a working model for the responses of seeds to hydration–dehydration (Figure 11), and it has potential value for studies on seed priming and survival in the soil seed banks.

## 4. Materials and Methods

### 4.1. Seed Materials

The study was performed on non-dormant seeds of *Lolium perenne*, *Nicotiana tabacum*, and *Arabidopsis thaliana*.

### 4.2. Hydration–Dehydration Cycles and Sampling Scheme

*A. thaliana*, *L. perenne*, and *N. tabacum* seeds were subjected to continual hydration–dehydration cycles. *A. thaliana*, *L. perenne*, and *N. tabacum* seeds were soaked in water at 25 °C for 2, 2, and 3 d, respectively. The surface water was then removed, and the seeds were dried over an excess of silica gel in a sealed bottle at laboratory temperature for 5 d. *A. thaliana* and *N. tabacum* seeds were subjected to six hydration–dehydration cycles, and *L. perenne* seeds were subjected to seven hydration–dehydration cycles. At each hydration–dehydration cycle, the ability of the seeds to germinate was determined. At specified points of the selected hydration–dehydration cycles, the lipids in the *A. thaliana* seeds were profiled.

### 4.3. Seed Germination

Each germination assay was conducted on four replicate groups of seeds. Each *A. thaliana*, *L. perenne*, and *N. tabacum* germination assay was performed using 40, 30, and 40 seeds, respectively. *A. thaliana* seeds were surface sterilized for 2 min using a 5% hypochlorite solution, then the seeds were rinsed twice with sterilized water. The sterilized seeds were sown in a Petri dish containing 0.7% agar-solidified Murashige and Skoog medium containing 1% sucrose, then the seeds were kept at 4 °C for 2 d. The *L. perenne* and *N. tabacum* seeds were sown directly in a Petri dish containing 1% agar-solidified medium. The seed germination tests were performed at 25 °C with light applied at 120 μmol m^−2^ s^−1^ light with a 16 h light 8 h dark cycle. Seed germination was scored when radicles more than 2 mm long had emerged from 3 d, 3 d, and 4 d until 30 d after sowing for *A. thaliana*, *L. perenne*, and *N. tabacum*, respectively.

### 4.4. Lipid Extraction, Electrospray Ionization Tandem Mass Spectrometry Analysis, and Data Processing

Lipid extraction and quantification procedures, as detailed previously, were used with minor modifications [31,38]. Lipids were extracted from five replicate aliquots of *A. thaliana* seeds per treatment, and each aliquot had a fresh weight of 20–30 mg. Powder from the ground seeds was immediately transferred to 3 mL of isopropanol containing 0.01% butylated hydroxytoluene at 75 °C to inhibit lipolytic activity. Each sample was extracted for 2 d with agitation, in 4 mL of a 2:1 mixture of chloroform and methanol containing 0.01% butylated hydroxytoluene. Each sample was extracted with the chloroform/methanol mixture three times, each time with agitation for 1 week. The remaining seed tissue was heated to 105 °C overnight, and then weighed. The weights of extracted and dried tissue were added together and defined as the dry weight of each sample. The lipid samples were analyzed using a triple quadrupole tandem mass spectrometer in electrospray ionization mode. The lipids in each class were quantified in comparison to the two internal standards of that class, using a correction curve determined between standards [31]. A Q-test was performed on the total amounts of lipids in each head-group class for the five replicates, and outlying data were then removed. One-way analysis of variance (ANOVA) and Fisher’s least significant difference (LSD) method were performed. Correlation coefficient analyses were performed using the Pearson method, using SPSS 19.0 software. The double bond index (DBI) was calculated using the formula

DBI = (Σ[Nb × mol % lipid])/100(1)

N is the number of double bonds in each molecular species. The ACL was calculated using the formula

ACL = (Σ[NC × mol % lipid])/100(2)

NC is the number of acyl carbon atoms in each molecular species.

## Figures and Tables

**Table 1:** Changes in the double bond index (DBI) for the total polar lipids in the *A. thaliana* seeds during the hydration–dehydration cycles.

Double Bond Index (DBI)				
C	3H	3D	4H	6D
3.05 ± 0.02 ^c^	3.05 ± 0.02 ^c^	3.13 ± 0.03 ^b^	3.02 ± 0.04 ^c^	3.31 ± 0.02 ^a^

The treatment was the same as that described in Figure 3. Each value is mean ± SD (*n* = 4 or 5). Values in the same row with different letters indicate that values are significantly different (*p* < 0.05).

## Supplementary Materials

Supplementary materials can be found at http://www.mdpi.com/1422-0067/19/5/1417/s1. Table S1: Glycerolipids analyzed in this study. R1 or R2, fatty acid. Cho, choline. EA, ethanolamine. Ser, Serine. Gro, glycerol. Ino, inositol. Gal, galactosyl. Cycled-P, PO^4−^; TableS2: Amounts of membrane lipids in each head-group class and the total membrane lipid contents of the *A. thaliana* seeds during the hydration–dehydration cycles. C: control (dry seeds), 3H: hydration part of the third hydration–dehydration cycle, 3D: dehydration part of the third hydration–dehydration cycle, 4H: hydration part of the fourth hydration–dehydration cycle, 6D: dehydration part of the sixth hydration–dehydration cycle. DW, Dry weight. TPL, Total polar lipid. Values in the same row with different letters are significantly different (*p* < 0.05). Each value is means ± SD (*n* = 4 or 5). * The PA contents were published previously [34]; Table S3: Compositions of the membrane lipids in each head-group class and the total membrane lipid contents of *A. thaliana* seeds during the hydration–dehydration cycles. The treatment was the same as that described in Table S2. Values in the same row with different letters are significantly different (*p* < 0.05). Each value is means ± SD (*n* = 4 or 5); Table S4: Acyl chain lengths of the membrane lipids in the *A. thaliana* seeds during the hydration–dehydration cycles. The treatment was the same as that described in Table S2. Each value is mean ± SD (*n* = 4 or 5). Values in the same row with different letters are significantly different (*p* < 0.05); Table S5: Lyso-phospholipid contents of the *A. thaliana* seeds during the hydration–dehydration cycles. The treatment was the same as that described in Table S2. Each value is mean ± SD (*n* = 4 or 5). Values in the same row with different letters are significantly different (*p* < 0.05).

## Abbreviations

PG	phosphatidylglycerol
PC	phosphatidylcholine
PE	phosphatidylethanolamine
PI	phosphatidylinositol
PS	phosphatidylserine
PA	phosphatidic acid
MGDG	monogalactosyldiacylglycerol
DGDG	digalactosyldiacylglycerol
DAG	diacylglycerol
ER	endoplasmic reticulum
DBI	double bond index
ACL	acyl chain lengths
