# Supplementary material for: Turnover of Glycerolipid Metabolite Pool and Seed Viability
Source: Int J Mol Sci. 2018 May 9;19(5):1417. doi: 10.3390/ijms19051417 (PMC5983817; doi:10.3390/ijms19051417)
Supplement: Supplementary file 1 [file ijms-19-01417-s001.pdf]

Supporting Information

Table S1. Glycerolipids analyzed in this study.

Glycerolipids	Abbreviation	Structure
Diacylglycerol	DAG	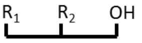
Phosphatidic acid	PA	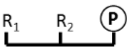
Phosphatidylcholine	PC	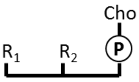
Phosphatidylethanolamine	PE	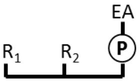
Phosphatidylserine	PS	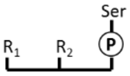
Phosphatidylglycerol	PG	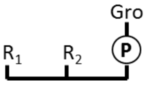
Phosphatidylinositol	PI	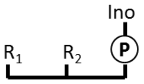
Monogalactosyldiacylglycerol	MGDG	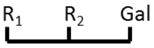
Digalactosyldiacylglycerol	DGDG	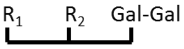

R1 or R2, fatty acid. Cho, choline. EA, ethanolamine. Ser, Serine. Gro, glycerol. Ino, inositol. Gal, galactosyl. Cycled-P, PO^{4-}.

Table S2. Amounts of membrane lipids in each head-group class and the total membrane lipid contents of the *A. thaliana* seeds during the hydration–dehydration cycles.

Lipid class	Lipid/dry weight (nmol/mg DW)				
	C	3H	3D	4H	6D
DGDG	0.76 ± 0.06^{a}	0.59 ± 0.04^{b}	0.36 ± 0.06^{d}	0.65 ± 0.03^{b}	0.44 ± 0.04^{c}
MGDG	0.29 ± 0.02^{a}	0.27 ± 0.02^{a}	0.06 ± 0.02^{d}	0.23 ± 0.01^{b}	0.11 ± 0.03^{c}
PG	0.17 ± 0.01^{b}	0.20 ± 0.02^{a}	0.02 ± 0.00^{c}	0.21 ± 0.01^{a}	0.00 ± 0.00^{c}
PC	3.24 ± 0.24^{c}	5.71 ± 0.31^{a}	0.12 ± 0.02^{d}	5.10 ± 0.16^{b}	0.04 ± 0.01^{d}
PE	1.46 ± 0.06^{b}	3.41 ± 0.24^{a}	0.12 ± 0.02^{c}	3.42 ± 0.19^{a}	0.10 ± 0.03^{c}
PI	3.56 ± 0.26^{b}	3.59 ± 0.33^{b}	0.93 ± 0.12^{c}	5.22 ± 0.34^{a}	0.55 ± 0.07^{d}
PS	0.08 ± 0.02^{c}	0.18 ± 0.06^{b}	0.06 ± 0.02^{cd}	0.23 ± 0.03^{a}	0.04 ± 0.01^{d}
PA*	1.37 ± 0.18^{b}	0.29 ± 0.04^{c}	1.77 ± 0.26^{a}	0.35 ± 0.03^{c}	1.87 ± 0.12^{a}
TPL	11.23 ± 0.90^{c}	14.72 ± 0.28^{b}	3.46 ± 0.48^{d}	15.52 ± 0.53^{a}	3.21 ± 0.25^{d}
DAG	30.39 ± 1.10^{bc}	21.28 ± 0.31^{d}	32.42 ± 4.59^{b}	27.69 ± 2.54^{c}	36.79 ± 3.76^{a}
TPL + DAG	41.62 ± 0.91^{a}	36.11 ± 1.06^{b}	35.88 ± 5.06^{b}	43.21 ± 2.75^{a}	40.00 ± 3.93^{ab}

C: control (dry seeds), 3H: hydration part of the third hydration–dehydration cycle, 3D: dehydration part of the third hydration–dehydration cycle, 4H: hydration part of the fourth hydration–dehydration cycle, 6D: dehydration part of the sixth hydration–dehydration cycle. DW, Dry weight. TPL, Total polar lipid. Values in the same row with different letters are significantly different ($p < 0.05$). Each value is means ± SD (n = 4 or 5). * The PA contents were published previously. [34]

Table S3. Compositions of the membrane lipids in each head-group class and the total membrane lipid contents of *A. thaliana* seeds during the hydration–dehydration cycles.

Lipid class	Lipids (mol %)				
	C	3H	3D	4H	6D
DGDG	6.83 ± 0.63^{c}	4.13 ± 0.22^{d}	10.39 ± 0.56^{b}	4.19 ± 0.22^{d}	13.67 ± 0.70^{a}
MGDG	2.55 ± 0.23^{b}	1.88 ± 0.10^{c}	1.81 ± 0.21^{c}	1.47 ± 0.13^{c}	3.26 ± 0.76^{a}
PG	1.52 ± 0.06^{a}	1.38 ± 0.10^{b}	0.44 ± 0.07^{c}	1.34 ± 0.02^{b}	0.11 ± 0.08^{d}
PC	28.84 ± 0.75^{c}	39.63 ± 0.67^{a}	3.45 ± 0.87^{d}	32.90 ± 1.51^{b}	1.34 ± 0.29^{e}
PE	13.62 ± 0.70^{c}	23.26 ± 0.38^{a}	3.50 ± 0.25^{d}	21.99 ± 0.62^{b}	3.00 ± 0.84^{d}
PI	31.72 ± 0.70^{b}	24.91 ± 1.42^{d}	26.95 ± 0.43^{c}	33.58 ± 1.30^{a}	16.96 ± 1.50^{e}
PS	0.73±0.15^{c}	1.26± 0.22^{b}	1.76± 0.31^{a}	1.49± 0.16^{ab}	1.22±0.19^{b}
PA	12.13±0.64^{c}	2.05±0.37^{d}	51.00±0.76^{b}	2.26± 0.15^{d}	58.35±4.94^{a}

The treatment was the same as that described in Table S2.Values in the same row with different letters are significantly different ($p < 0.05$). Each value is means ± SD (n = 4 or 5).

Table S4. Acyl chain lengths of the membrane lipids in the *A. thaliana* seeds during the hydration–dehydration cycles.

**Lipid class**	**Acyl chain length (carbon)**				
	**C**	**3H**	**3D**	**4H**	**6D**
DGDG	35.39 ± 0.05[b]	35.43 ± 0.03[ab]	35.44 ± 0.04[ab]	35.46 ± 0.03[a]	35.48 ± 0.05[a]
MGDG	34.56 ± 0.05[c]	35.35 ± 0.04[b]	35.42 ± 0.22[b]	35.49 ± 0.07[b]	35.58 ± 0.06[a]
PG	33.97 ± 0.06	34.02 ± 0.06	34.25 ± 0.20	34.13 ± 0.08	34.00 ± 1.21
PC	35.73 ± 0.03[b]	35.74 ± 0.04[b]	35.99 ± 0.11[a]	35.79 ± 0.02[b]	35.97 ± 0.09[a]
PE	35.65 ± 0.01[b]	35.63 ± 0.02[b]	35.93 ± 0.11[a]	35.72 ± 0.02[b]	35.94 ± 0.14[a]
PI	34.68 ± 0.02[d]	34.71 ± 0.00[cd]	34.93 ± 0.03[b]	34.73 ± 0.01[c]	35.00 ± 0.02[a]
PS	40.94 ± 0.37[c]	39.63 ± 0.18[d]	41.97 ± 0.29[b]	39.72 ± 0.17[d]	42.61 ± 0.41[a]
PA	35.41 ± 0.02[b]	35.46 ± 0.03[a]	35.51 ± 0.02[a]	35.50 ± 0.06[a]	35.49 ± 0.03[a]
Total polar lipid	34.93 ± 0.03[d]	35.26 ± 0.03[c]	35.35 ± 0.05[b]	35.29 ± 0.03[c]	35.42 ± 0.03[a]
DAG	35.51 ± 0.00[b]	35.56 ± 0.03[a]	35.44 ± 0.02[c]	35.59 ± 0.01[a]	35.30 ± 0.04[d]

The treatment was the same as that described in Table S2. Each value is mean ± SD (n = 4 or 5). Values in the same row with different letters are significantly different ($p < 0.05$).

Table S5. Lyso-phospholipid contents of the *A. thaliana* seeds during the hydration–dehydration cycles.

**Lipid class**	**Lipid/dry weight (nmol/mg DW)**				
	**C**	**3H**	**3D**	**4H**	**6D**
LysoPG	0.021 ± 0.003[a]	0.013 ± 0.005[b]	0.010 ± 0.005[bc]	0.007 ± 0.002[c]	0.006 ± 0.004[c]
LysoPC	0.163 ± 0.020[a]	0.099 ± 0.014[b]	0.009 ± 0.004[d]	0.071 ± 0.004[c]	0.005 ± 0.003[d]
LysoPE	0.046 ± 0.002[a]	0.042 ± 0.005[a]	0.005 ± 0.001[b]	0.043 ± 0.005[a]	0.006 ± 0.002[b]

The treatment was the same as that described in Table S2. Each value is mean ± SD (n = 4 or 5). Values in the same row with different letters are significantly different ($p < 0.05$).

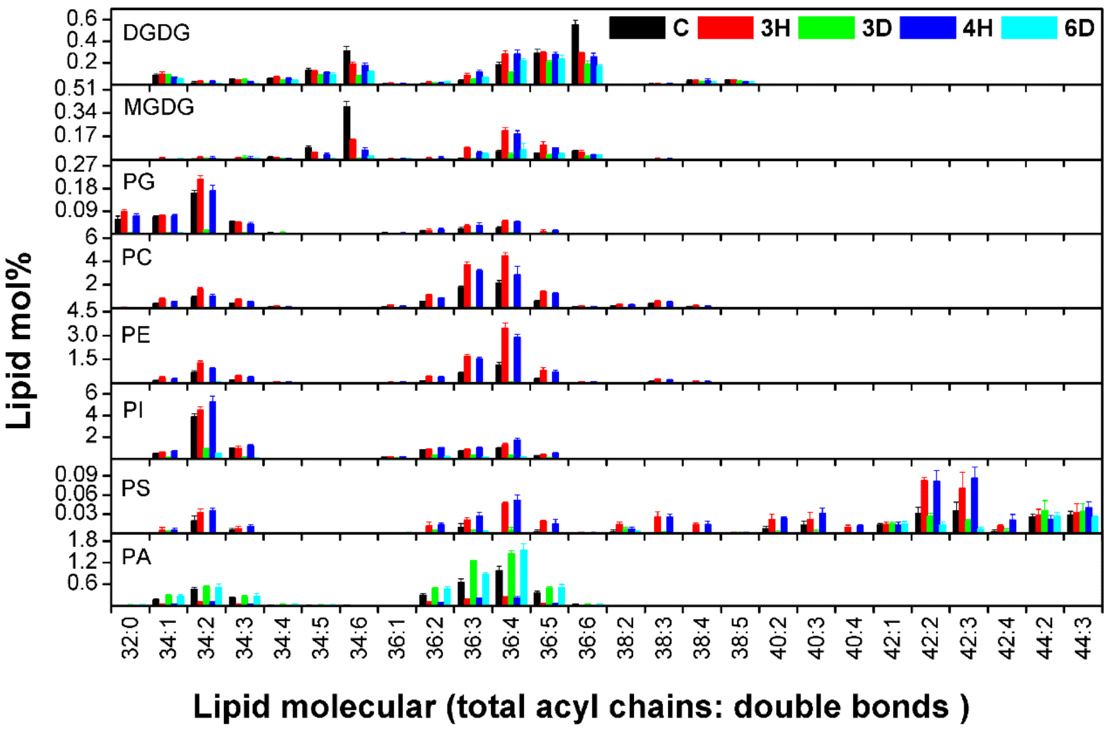

Figure S1. Compositions of the lipid molecular species in *A. thaliana* seeds during the hydration–dehydration cycles. The treatment was the same as that described in Table S2. Each value is mean ± SD (n = 4 or 5).

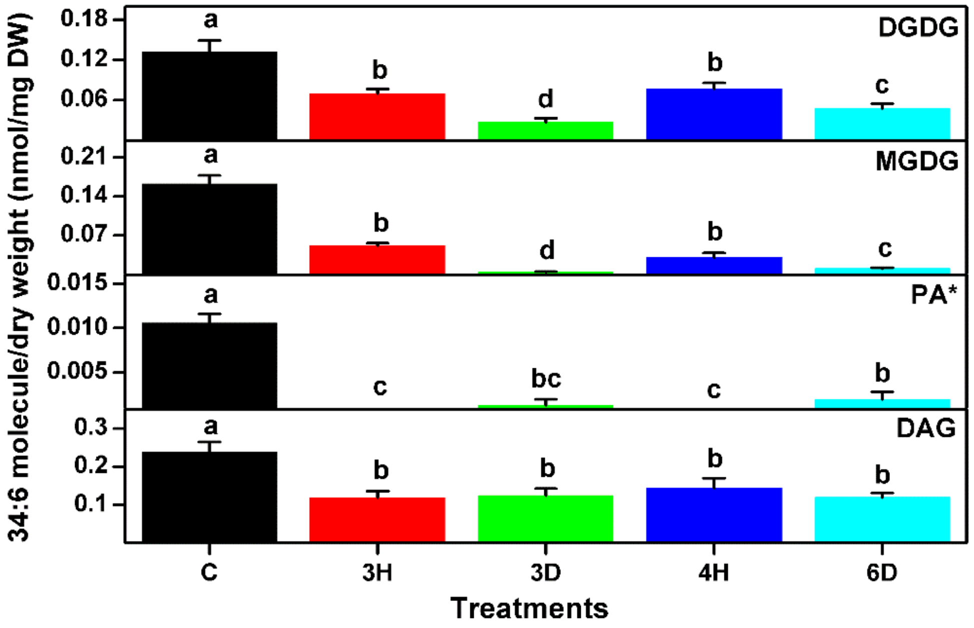

Figure S2. Effects 34:6 molecular species contents of the *A. thaliana* seeds during the hydration–dehydration cycles. The treatment was the same as that described in Table S2. DW, dry weight. Each value is mean ± SD (n = 4 or 5). Bars for the same lipid class with different letters indicate that the values were significantly different ($p < 0.05$). * The PA 34:6 molecular species contents were published previously. [34]
